# Face perception influences the programming of eye movements

**DOI:** 10.1038/s41598-018-36510-0

**Published:** 2019-01-24

**Authors:** Louise Kauffmann, Carole Peyrin, Alan Chauvin, Léa Entzmann, Camille Breuil, Nathalie Guyader

**Affiliations:** 1 Univ. Grenoble Alpes, CNRS, Grenoble INP, GIPSA-lab, 38000 Grenoble, France; 2 Univ. Grenoble Alpes, Univ. Savoie Mont Blanc, CNRS, LPNC, 38000 Grenoble, France

## Abstract

Previous studies have shown that face stimuli elicit extremely fast and involuntary saccadic responses toward them, relative to other categories of visual stimuli. In the present study, we further investigated to what extent face stimuli influence the programming and execution of saccades examining their amplitude. We performed two experiments using a saccadic choice task: two images (one with a face, one with a vehicle) were simultaneously displayed in the left and right visual fields of participants who had to initiate a saccade toward the image (Experiment 1) or toward a cross in the image (Experiment 2) containing a target stimulus (a face or a vehicle). Results revealed shorter saccades toward vehicle than face targets, even if participants were explicitly asked to perform their saccades toward a specific location (Experiment 2). Furthermore, error saccades had smaller amplitude than correct saccades. Further analyses showed that error saccades were interrupted in mid-flight to initiate a concurrently-programmed corrective saccade. Overall, these data suggest that the content of visual stimuli can influence the programming of saccade amplitude, and that efficient online correction of saccades can be performed during the saccadic choice task.

## Introduction

Faces are very salient visual stimuli for humans and our visual system has developed efficient mechanisms to preferentially detect and process them. A particular status of face stimuli is supported by a large number of studies, which consistently showed that faces elicit fast and characteristic brain and behavioral responses, as compared to other categories of visual stimuli^1–6^. Eye-tracking studies for example showed that during free exploration of complex scenes containing faces, observers immediately direct their gaze toward them and spend a lot of the exploration time looking at them^7–11^.

Recent studies have adopted a paradigm in which eye movements are used as a behavioral response to investigate the speed of face processing. In this paradigm, called saccadic choice task, two images are simultaneously displayed on a screen in the left and right visual fields. One image contains a target stimulus (e.g., a face) and the other one a distractor (e.g., a vehicle). Participants have to perform a saccade as fast as possible toward the target stimulus (e.g., a face^3,12–15^). Although this task requires multiple processes (i.e. simultaneous processing of two images, a categorical decision, programming and execution of an eye movement toward the target), it has been shown that human observers are able to initiate accurate saccadic responses toward a face with extremely short latencies of just 100–110 ms^3^. Such latencies are barely above the earliest latencies observed for reflexive saccades elicited by the appearance of an image or a simple dot stimulus in the periphery (see for example^3,15–17^), highlighting the remarkable speed of face processing. Furthermore, these studies also reported that participants made more error saccades (i.e. saccades toward the distractor) when the distractor was a face than when it was another stimulus (e.g., a vehicle). This suggests that fast saccades toward faces tend to be automatic and can be beyond voluntary control^4,18^, see also^19^. Further studies using the same saccadic choice task revealed that the bias for face stimuli persists even if they are presented at large eccentricities (up to 80°)^13^ or if stimuli are filtered so that only coarse, low-spatial frequency information remains in the stimulus^14^.

Overall, these eye-tracking data suggest that faces contain specific information that influences the programming of saccades by triggering extremely fast and automatic orienting responses toward them. However, these studies only focused on the analysis of saccade latency and accuracy. These parameters inform about the speed of processes involved in the task and the ability to select (suppress) an appropriate (inappropriate) response, respectively. Yet, other saccade parameters would be relevant to further inform about saccade programming. For example, studies investigating the programming of saccades also analyzed their precision (i.e. the distance between their ending point and the target point) or their amplitude (i.e. the distance between the starting and ending point of a saccade). In the classical view, the amplitude of a saccade is thought to be programmed at its onset and would not be influenced by new visual information once initiated, thereby informing about saccade programming prior to its execution (although small online corrections can be observed based on internal feedbacks^20^. In the absence of a visual distractor, saccades toward a peripheral target stimulus are accurate in terms of spatial precision - although it is frequently observed that saccades land between the starting and target position^21–23^. However, when a distractor is presented on the saccade trajectory to a target, the saccade tends to land at an intermediate location between the two stimuli. More precisely, the saccade would land beyond (overshoot) or in front of (undershoot) the target if the distractor is also presented beyond or before the target, respectively e.g.^24^, see for a review ^25,26^. Furthermore, the landing position of the saccade – toward one stimulus or the other – can be modulated according to stimulus characteristics, such as their size (e.g., saccades tend to land closer to the biggest stimulus^27^) or task constraints (e.g., saccades tend to land closer to one stimulus if it has been defined as the target but would land in between the two stimuli if there is no specific target to reach^28,29^). This phenomenon, known as ‘saccade averaging’, is interpreted as resulting from the weighted average of activity of neurons coding for each stimulus location on a common saccade map. These studies thus indicate that the presence and properties of a distractor stimulus can influence the programming of saccade amplitude toward a target. However, whether the content of the target and distractor stimuli in the context of a saccadic choice task can also impacts programming of saccade amplitude has, to our knowledge, never been addressed.

Other studies also reported a modulation of saccade amplitude according to the accuracy of the saccadic response^30–33^. For example, using a visual search task, in which participants were presented with a display of stimuli and had to initiate a saccade toward one of them based on its color, McPeek *et al*.^30^ observed that in a small proportion of trials, participants made error saccades which were characterized by small amplitudes. Similarly, using an anti-saccade task (i.e. perform a saccade in the direction opposite to the appearance of a target), Weber *et al*. (1997) observed that error saccades were often hypometric. Interestingly, in these studies, hypometric initial error saccades were generally followed by a second saccade bringing the gaze to the correct target location after a very short delay (0–100 ms), well below the range of normal saccade latencies (120–200 ms). These short inter-saccadic intervals between two consecutive saccades were taken as evidence that the programming of the second saccade occurred in parallel and interfered with the initial one, resulting in a reduction of the initial saccade’s amplitude^30,31,33,34^, see also^35^. These studies led to propose that two saccades oriented toward different goals can be programmed concurrently on a common saccade map and that the different programs would compete with each other. The program winning the competition would be executed, but could still be influenced and even curtailed once initiated by a concurrent program, resulting in a reduction of the first saccade amplitude and the execution of a second saccade after a very short delay^30,34,36–39^. It is likely that the saccadic choice task also involves parallel programming of two saccades (i.e. one toward each image) competing and interfering with each other. The examination of saccade amplitude according to response accuracy, but also of the characteristics (e.g., latency, amplitude) of saccades following an error would thus allow to test this hypothesis. Importantly, it would also allow to test for potential differences between parallel programming of saccades according to the content of their target, which has never been investigated so far. It is for example possible that a saccade program toward a face stimulus interferes more strongly with a concurrently programmed saccade toward a vehicle than the other way around.

The present study thus aimed to further investigate the extent to which the content of complex visual stimuli, in particular the face content, influence saccade programming and execution. To this end, we performed two experiments using a saccadic choice task in which images of faces and vehicles were presented simultaneously to participants in their right and left visual fields. In Experiment 1, we used the same saccadic choice task as in previous studies^3,14^. Participants were asked to perform saccades as fast as possible toward the side of the display containing the target stimulus (a face or a vehicle). Whereas previous studies only analyzed saccade accuracy and latency, we focused in the present one on the amplitude of saccades, as reflecting an aspect of saccade programming prior to their execution allowing to better understand the mechanisms underlying saccadic choices. The amplitude of saccades was analyzed taking into account the target category (face vs. vehicle), and the accuracy of saccadic responses (correct saccades toward the target vs. error saccade toward the distractor). In Experiment 2, we used the same design but this time, participants were explicitly asked to perform their saccades toward a fixation cross added in the center of the images. This allowed us to better estimate the precision of saccades with respect to the target cross to reach, and thus to quantify how saccade amplitude was modulated by the content of visual stimuli and the accuracy of saccadic responses.

Given previous reports suggesting that face stimuli elicit fast and involuntary saccades toward them, we expected that the presence of a face distractor would also impact amplitude of saccades directed toward another target stimulus. Furthermore, based on previous findings (e.g., McPeek *et al*.^30^, we expected error saccades to be shorter than correct saccades, suggesting their modulation by a concurrently programmed corrective saccade. Finally, we examined the possibility that concurrent programming of saccades was also influenced by the content of their targets.

## Experiment 1

### Materials and Methods

#### Participants

Twenty-four participants (three females; mean age ± SD = 25.5 ± 6.26 years) with normal or corrected-to-normal vision, recruited from University Grenoble Alpes, took part in the experiment. They all came twice to complete two experimental sessions, one with faces as target stimuli and one with vehicles as target stimuli. All participants gave their informed written consent before participating in the study, which was carried out in accordance with the Code of Ethics of the World Medical Association (Declaration of Helsinki) for experiments involving humans and was approved by the ethic committee of University Grenoble Alpes.

#### Stimuli

Stimuli were 240 colored images taken among the stimuli used by Crouzet *et al*.^3^ and chosen from Corel Stock Photo Libraries (Corel Photo stock library 1996. Ottawa, Ontario, Canada) widely used in literature for visual recognition. There were 120 images of human faces and 120 images of vehicles of various size and taken from different viewpoints. Out of the 120 images used for each category, there were 42 images of faces and 24 images of vehicles for which the main object was symmetrical around the vertical midline. However, this difference did not impact our results (see Supplementary Analysis S1 and Table S1). Stimuli were carefully chosen so that faces and vehicles had on average the same spatial position and size. For each image, the main object, i.e. face or vehicle, was manually delineated using a rectangle box (size 8.35 × 7.96°). We then computed the center of the box and ensured that no significant difference was observed for the mean position or size of objects between faces and vehicles images (mean center X_face_ = 5.86°; mean center X_vehicle_ = 5.89°; t_238_ = −0.25; p = 0.8; mean center Y_face_ = 5.86°; mean center Y_vehicle_ = 5.83°; t_238_ = 0.21; p = 0.84; zero being the top left corner of the image). Stimuli (sized 300 × 300 pixels) subtended 11 × 11° of visual angle at a viewing distance of 60 cm. For each category, 10 images were used for training and the remaining 110 images were used in the main experiment. It should be noted that some of these images were previously used in Guyader *et al*.^14^.

#### Procedure

Stimuli were displayed using the Softeye software^40^ against a gray background (luminance of 128 on a 256 gray-level scale) on a 21-inch CRT monitor with a spatial resolution of 1024 × 768 pixels, a refresh rate of 85 Hz and a mean gray luminance of 68 cd/m^2^. Participants were seated 60 cm away from the display. Participants’ head was stabilized by a chin- and a forehead rest. Eye movements were recorded using an Eyelink 1000 eye-tracker (SR Research) with a sampling rate of 1000 Hz and a nominal spatial resolution of 0.01 degrees of visual angle. Only one eye was recorded using the “pupil-corneal reflexion” mode (right eye in 16 out of 24 participants). The Eyelink software automatically detected saccades with the following thresholds: speed >30 degrees/s, acceleration >8000 degrees/s^2^, and saccadic displacement >0.15 degrees. Fixations were detected when the pupil was visible, and no saccade was in progress. Blinks were detected during partial or total occlusion of the pupil. Each session was preceded by a calibration procedure during which participants had to orient their gaze toward nine separate targets appearing sequentially in a 3 × 3 grid that occupied the entire display. A drift correction was carried out every ten trials and a new calibration was done in the middle of the experiment and when the drift error was above 0.5°.

**Figure 1:**
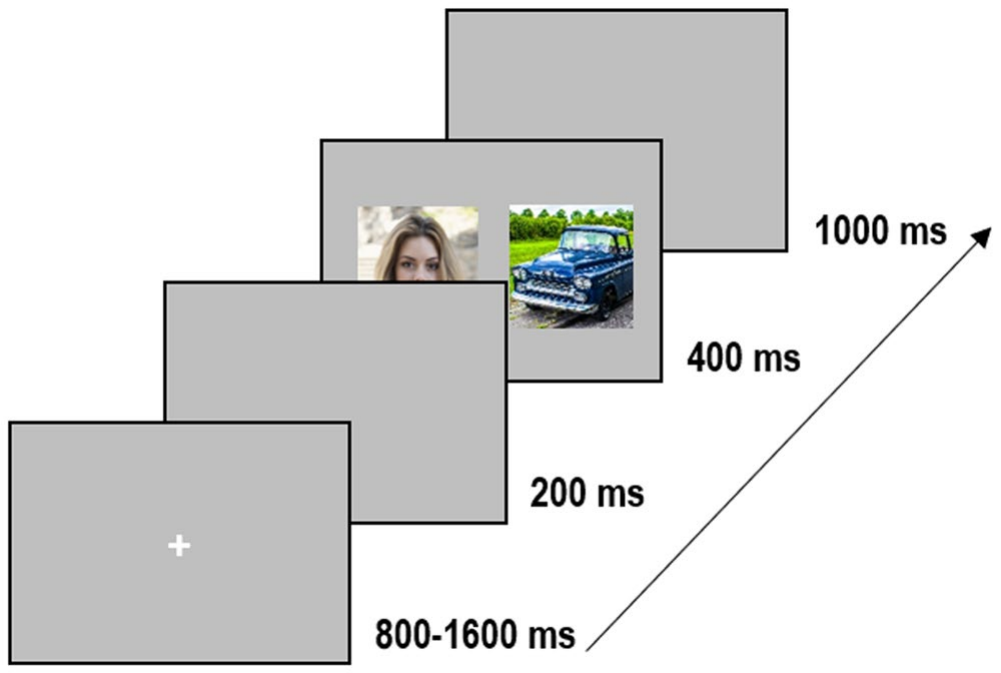
Time course of a trial. Participants had to fixate a central cross (800–1600 ms). After a gap of 200 ms, two images were displayed in the left and right visual fields (image centers were at 7.6° of eccentricity) during 400 ms, followed by a gray screen during 1000 ms. In each trial, participants had to perform a saccade toward the image containing the target stimulus (face or vehicle).

All participants underwent two experimental sessions on two different days (separated by less than two weeks), one session for which the targets were human faces (and the distractors were vehicle images) and the other one for which the targets were images containing vehicles (the distractors were human face images). The order of sessions was counterbalanced between participants. The procedure and task were exactly the same as in Crouzet *et al*.^3^ and Guyader *et al*.^14^. For each session, a trial started with a white fixation cross subtending 0.73° of visual angle, displayed centrally for 800 to 1600 ms (duration sampled from a uniform distribution) and followed by a gap (mean gray-level screen) of 200 ms. Following the gap, two images (a target and a distractor) were simultaneously displayed on the left and the right of the central fixation cross for 400 ms. The center of each image was lateralized at 7.6° from the center of the screen. The inter-trial interval was fixed at 1000 ms (Fig. 1). Participants had to make a saccade as fast as possible toward the target image. There were 240 trials in each session, each image being seen twice, on the left and the right side, randomly. Participants completed a training session comprising 10 trials prior to the experiment in order to get familiarized with the stimuli and the task. The experiment lasted approximately 15 minutes in each session.

#### Data analyses

The accuracy (% error), latency (in milliseconds from the onset of stimuli - also called saccadic reaction time, SRT) and amplitude (distance between the positions of the start and the end of saccades, in degrees of visual angle) of the first saccade were analyzed. Saccades were automatically detected by the Eyelink software. When applicable, we also examined the SRT and amplitude of the second saccade. We also extracted the peak speed of saccades. Trials in which a blink occurred during stimulus presentation or for which SRT was shorter than 50 ms were discarded from the analysis, in order to avoid including express saccades not related to the processing of stimuli from the analysis (see also Supplementary Analysis S2, Supplementary Fig. S1 and Tables S2 and S3). This resulted in removing 1.49% of the trials. Analyses were performed using Matlab (MathWorks, Natick, MA) and Statistica 10.0 software (Statsoft, Tulsa, USA) after ensuring that the respective assumptions were met for each analysis (e.g., ANOVA, t tests). Effect sizes were estimated by calculating partial eta-squared (η_p_^2^). The significance level of tests was set at α = 0.05 and Bonferroni-corrected p-values are reported for pairwise comparisons. Data and material can be made available upon request to the authors.

### Results

#### Accuracy

We performed a paired t-test on mean error rates with Target Category (Face, Vehicle) as within-subject factor. Results revealed that participants made significantly more error saccades when the target stimulus was a vehicle (i.e. face distractor mER ± SD: 23.37 ± 10.62%) than when it was a face (i.e. vehicle distractor; 12.46 ± 6.40%, t_23_ = 5.17, p < 0.001).

#### Latency and Amplitude of the first saccade

ANOVAs with Target Stimulus (Face, Vehicle) and Saccade Accuracy (Correct, Error) as within-subject factors were performed on mean SRT of the first saccade (in ms) and saccade amplitude (in degrees).

**Figure 2:**
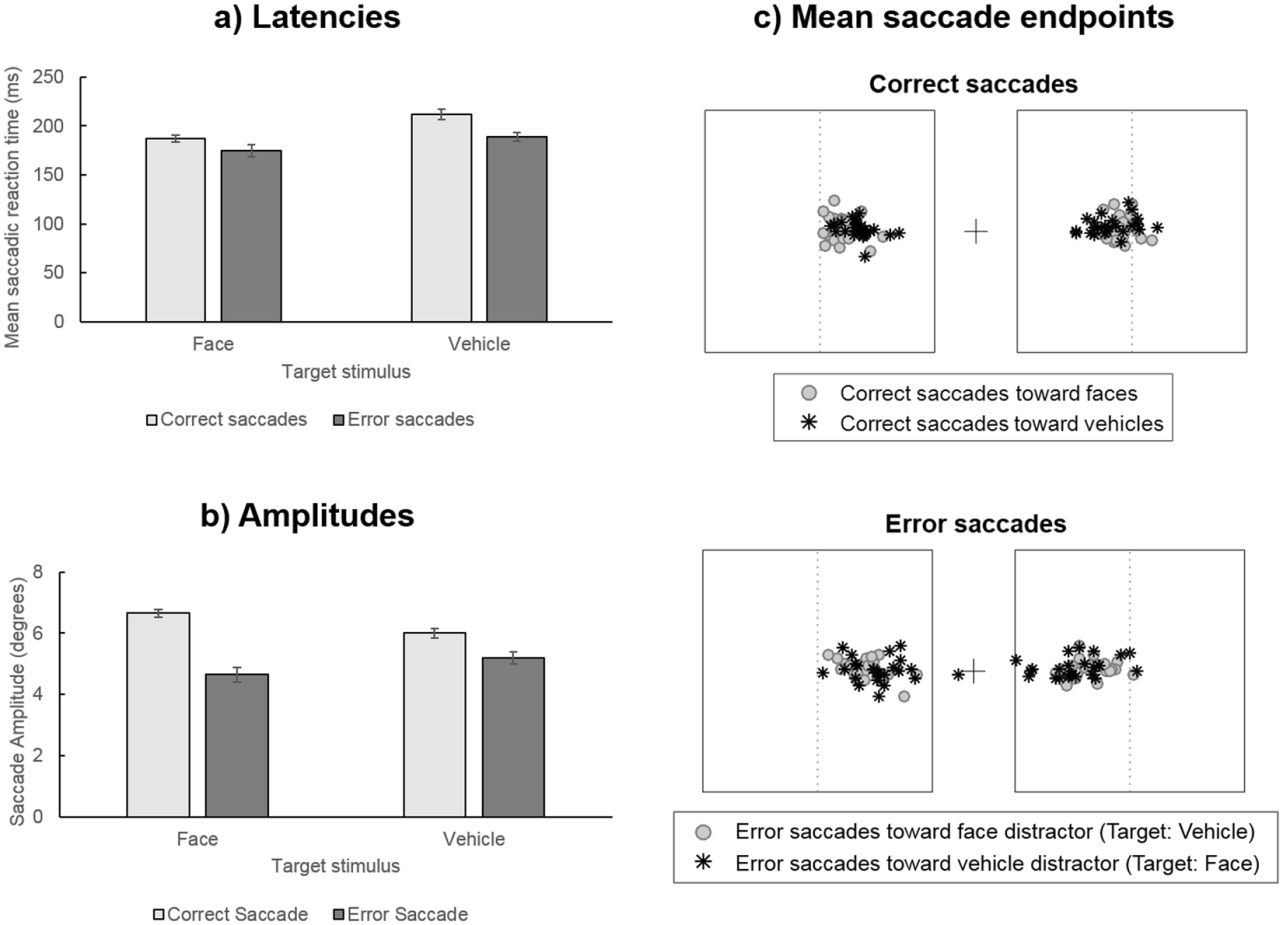
(**a**) Mean saccadic reaction times (in milliseconds) and (**b**) mean amplitudes (in degrees) of correct (light grey) and error saccades (dark grey), according to the target category (face, vehicle). Error bars indicate standard error. (**c**) Mean ending points of correct (top) and error saccades (bottom) of each participant according to the target category (face, vehicle). The black boxes represent the edges of images, the central cross represents the fixation point and the dotted lines indicate the middle of images.

The ANOVA performed on mean SRT (Fig. 2a) revealed a main effect of Target Category (F_1,23_ = 18.21, p < 0.001, η_p_^2^ = 0.442) and a main effect of saccade Accuracy (F_1,23_ = 34.37, p < 0.0001, η_p_^2^ = 0.599). Participants initiated saccades faster when the target stimulus was a face (183 ± 21 ms) than when it was a vehicle (201 ± 22 ms) and they were slower to initiate correct (200 ± 19 ms) than incorrect saccades (184 ± 21 ms). There was a marginally significant interaction between Target Category and Saccade Accuracy (F_1,23_ = 3.33, p < 0.08, η_p_^2^ = 0.126), suggesting that the difference in latencies according to the target stimulus was more pronounced for correct (25 ms difference with 187 ± 17 ms for Face target and 212 ± 26 ms for Vehicle target) than error saccades (14 ms difference with 175 ± 30 ms for Face target and 189 ± 21 ms for Vehicle target).

The ANOVA performed on mean Saccade Amplitude (Fig. 2b) revealed no significant main effect of Target Category (F_1,23_ < 1, η_p_^2^ = 0.007) but there was a main effect of Saccade Accuracy (F_1,23_ = 142.1, p < 0.0001, η_p_^2^ = 0.861). Error saccades (4.92 ± 0.99°) were shorter than correct saccades (6.33 ± 0.60°). We also observed a significant interaction between Target Category and Saccade Accuracy (F_1,23_ = 51.67, p < 0.0001, η_p_^2^ = 0.692). Pairwise comparisons showed that correct saccades were larger when the target was a face than when it was a vehicle (faces: 6.66 ± 0.62°; vehicle: 6.00 ± 0.79°, p < 0.001) but this difference was reversed for error saccades, which were larger when the target was a vehicle (i.e. face distractor: 5.20 ± 96°) than when it was a face (i.e. vehicle distractor: 4.64 ± 1.15°, p < 0.005). This therefore indicates overall larger saccades directed toward faces (either as target or distractor) than vehicles. Furthermore, error saccades were shorter than correct saccades for both Target Categories (both ps < 0.0001). Figure 2c illustrates the mean ending locations of correct and error saccades for all participants on the display, according to the target category. As can be seen on this figure, error saccades were closer to the central fixation cross than correct saccades. Furthermore, error saccades toward face distractors were also closer to the center of the display than error saccades toward vehicle distractors.

#### Analysis of corrective saccades

**Figure 3:**
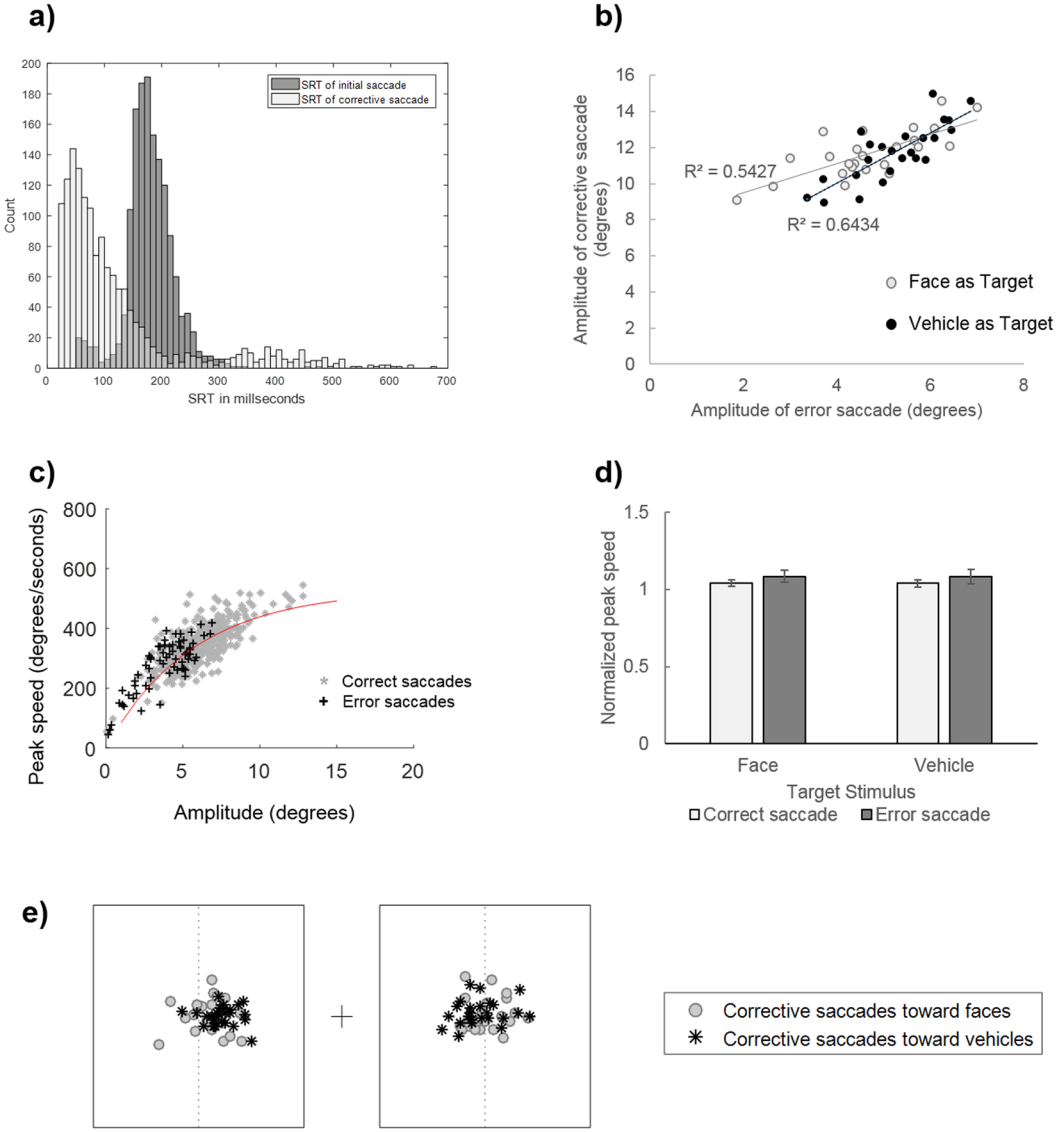
(**a**) Distributions of saccadic reaction times (SRT) of initial error saccades (dark grey) and of ensuing corrective saccades (light grey). (**b**) Correlations between the amplitude of error saccades and the amplitude of ensuing corrective saccades. (**c**) Example of main sequence for correct and error saccades of a participant. The red line corresponds to the exponential function fitted to correct responses. (**d**) Mean normalized peak speed of correct and error saccades, according to the category of the target stimulus. Error bars indicate standard error. (**e**) Mean ending points of corrective saccades of each participant according to the target category (face, vehicle). The black boxes represent the edges of images, the central cross represents the fixation point and the dotted lines indicate the middle of images.

We also examined whether error saccades, which represented 16.6% of the trials, were followed by corrective saccades. Second saccades were considered as corrective if their ending point was on the side of the display where the target was present (i.e. if saccades crossed the central fixation point). Using this criterion, 90.93% of saccades following an error were considered as corrective, and were used in subsequent analyses. Thus, the majority of error saccades were followed by a corrective saccade moving the gaze to the target image. Critically, these corrective saccades had very short latencies with a median SRT of 82 ms (min = 21 ms; max = 675 ms; mean = 124 ms, SD = 118 ms; cf. Fig. 3a), indicating that more than half of corrective saccades had latencies below 100 ms. Furthermore, as can be seen on Fig. 3a, the distributions of SRT for the initial error saccades and of the second corrective saccades have very distinct peaks. Such extremely short inter-saccadic intervals thus suggest that the second saccade was programmed in parallel to the execution of the initial error saccade^30,33–35,38,41^. We further investigated whether these corrective saccades were influenced by the Target Category (as observed for the first saccade in terms of proportion, latency and amplitude). To this end, we performed paired t-tests comparing the proportion, amplitude and latency of corrective saccades toward faces vs. vehicles. However, there was no significant difference between the proportion (t_23_ = 1.19, p = 0.25), latency (t_23_ = −0.58, p = 0.57) or amplitude (t_23_ = 1.25, p = 0.22) of corrective saccades.

As shown in the analysis of the amplitude of initial saccades, error saccades were smaller than correct saccades (Fig. 2b). This is also illustrated in Fig. 2c where the ending points of error saccades appear to be much closer to the center of the display, relative to correct saccades. This could suggest that error saccades were interrupted, to initiate a corrective saccade toward the target. In order to test this hypothesis, we first examined whether the corrective saccade compensated for the amplitude of the initial error saccade. Indeed, it is possible that two saccades toward each image are prepared in parallel at the beginning of each trial and both would be executed consecutively. In this case, the amplitude of the second saccade should not be linked to the amplitude of the first saccade because both the first and the second saccades would be planned from the fixation point. Alternatively, the second saccade could be programmed in order to compensate for the amplitude of the initial error saccade. In this case, its programming and hence its amplitude should compensate for the fact that the first saccade brought the eyes away from the target. This would be reflected by a positive correlation between the amplitudes of the error and corrective saccades. Results suggested the latter case, as we observed significant correlations between the mean amplitudes of initial and second saccades for both target conditions (Face as target: r = 0.74, p < 0.0001; Vehicle as target: r = 0.80, p < 0.0001), indicating that the higher the amplitude of error, the higher the amplitude of the corrective saccade toward the target (Fig. 3b,e).

#### Analysis of saccade kinematics

The previous analyses revealed that error saccades were hypometric relative to correct saccades, and followed by corrective saccades with very short latencies, suggesting their programming occurred in parallel with the first saccade. Furthermore, these analyses showed that the amplitude of saccades following an error increased with the amplitude of the error, suggesting that they were programmed to compensate for the error. We thus further tested whether the corrective saccade could also have interrupted the initial error one, which could explain the reduced amplitude of error relative to correct saccades. To this end, we examined saccades kinematics. Saccade kinematics are known to follow regularities characterized by an increase of peak speed with increasing amplitude of saccade. The relation between peak speed and saccade amplitude has been called the main sequence^42^. Simple two-parameter functions have been proposed to model the main sequence. If a saccade programmed to achieve a certain amplitude is interrupted in mid-flight, its kinematics should exhibit a violation of the main sequence, with a higher peak speed than the one predicted by its main sequence for a particular amplitude^30,43^. In order to address this question, we first extracted the main sequence (i.e. peak speed as a function of saccade amplitude) of correct saccades for each participant. We then compared the peak speed observed for error saccades to the peak speed predicted by the main sequence of correct saccade, given the saccade amplitude (see Fig. 3c and Supplementary Fig. S2). Predicted peak speed was calculated as follows: an exponential function was fitted (using EzyFit toolbox; Moisy, F http://www.fast.u-psud.fr/ezyfit/ V 2.45) to the main sequence (i.e. observed peak speed as a function of observed amplitude) of correct saccades for each participant irrespective of the Target category. The fitted function was:

$$PS(a)={c}_{1}(1-{e}^{-\frac{a}{{c}_{2}}})$$

With *PS* the peak speed (in degrees/s), *a* the amplitude (in degrees) and *c*_1_ and *c*_2_ the parameters of the model **(**in degrees/s and in degrees, respectively**)**. For each participant, a classical least-square fitting procedure was used to estimate the two parameters (mean R² = 0.69, SD = 0.21). For each trial, the 2-parameters function was used to estimate the predicted peak speed based on the saccade amplitude observed at this trial. This predicted peak speed was then used to normalize the observed peak speed of correct saccades and of error saccades followed by a corrective saccade (see Buonocore *et al*.^43^ for a similar procedure). Normalized peak speed higher than 1 indicates higher peak speed than what is predicted by the observed amplitude.

We then performed an ANOVA on normalized peak speed with Target Category (Face, Vehicle) and Saccade Accuracy (Correct, Error) as within-subject factors (see Fig. 3d). It should be noted that two subjects were excluded from this analysis due to aberrant fit parameters leading to biased estimation of predicted peak speed. Results revealed a main effect of Saccade Accuracy (F_1,21_ = 6.09, p < 0.05, η_p_^2^ = 0.225), suggesting a violation of the main sequence for error saccades: normalized peak speed of error saccades (1.08 ± 0.13) were higher than that of correct saccades (1.04 ± 0.07). There was no main effect of Target Stimulus, nor interaction with Saccade Accuracy (both F_s_ =<1, η_p_^2^ < 0.050).

### Discussion

Results of Experiment 1 replicated previous findings showing that participants make less errors and are faster to initiate saccades toward faces than toward vehicles^3,12–14^. Importantly, our results additionally revealed that saccades toward faces were also larger than saccades toward vehicles. As the position of target stimuli within the images was controlled for and given that all images were presented once in each visual field, this result cannot be explained by an overall larger eccentricity of faces relative to vehicles in the images. Furthermore, the average amplitude of correct saccades toward faces (6.66°) did not exceed the distance between the central fixation point and the center of lateral images (set at 7.6° of eccentricity), suggesting that they were not abnormally large but rather that saccades toward vehicles (mean amplitude: 6°) were short. This is illustrated in Fig. 2c in which the mean ending points of saccades on the display are plotted for all participants. This result therefore suggests that the content of the target/distractor image influences programming of saccade amplitude in the saccadic choice task. However, as the task did not involve any constraint on the ending point of saccades (i.e. participants were asked to perform a saccade “toward the image containing the target” and not toward a particular location in this image), the extent to which saccade amplitude is affected by the content of the target/distractor stimulus could not be precisely estimated. Indeed, it has been shown that when participant freely explore complex scenes containing faces, they preferentially look at them and tend to fixate at their center^10,11^. However, when faces are absent, the location of fixations is rather predicted by low-level salient features within the image^10^. It is thus possible that when the target was a face, participants preferentially directed their saccade toward the center of the face, whereas they did not necessarily reach the center of vehicles in images when this stimulus was defined as the target and ended their saccades before.

Another interesting finding of Experiment 1 was that amplitude of saccade was also modulated by the accuracy of the saccadic response, error saccades being much shorter than correct saccades (see Fig. 2b,c). Furthermore, these hypometric error saccades were generally followed by a second saccade bringing the gaze to the target image with extremely short inter-saccadic intervals from just 20 ms. This pattern of results was previously observed in eye-tracking studies involving a saccadic response such as visual search tasks, anti-saccade tasks or double-step paradigms^30,31,33,34^. They were taken as evidence that the programming of two saccades occurred in parallel and that the development of the second saccade program could curtail or even interrupt the initial one, resulting in a smaller amplitude. In order to disambiguate this question, we examined the kinematics of saccades, which revealed that error saccades deviated from the main sequence of correct saccades, with higher peak speed than what was predicted by their amplitude. This latter result thus rather supports the idea that error saccades were interrupted by the development and execution of a concurrent saccade program toward the target image. Critically, the amplitude of error saccades was also modulated by the content of their target, error saccades directed toward vehicle distractors being even shorter than error saccades directed toward face distractors. This could thus indicate that when an initial error saccade was made toward a distractor stimulus, the programming of a corrective saccade toward a face developed and interrupted the initially executed saccade earlier than corrective saccades toward a vehicle. However, we did not find evidence for a difference in the corrective saccade parameters according to the content of their target. Importantly, analyses of the amplitude of error and corrective saccades indicated that the amplitude of saccades following an error increased with the amplitude of the error. This suggests that they were programmed to compensate for the error saccade amplitude while it was being executed. However, as the task did not involve a specific location to reach that could serve as basis to estimate the precision of the corrective saccade, this question could not be further addressed in the present experiment.

Experiment 2 was thus designed to disambiguate the extent to which saccade amplitude was affected by the content of the target and distractor stimuli, as well as the extent to which saccades following an error compensated for the amplitude of the error, by constraining the ending location of saccadic responses. Design and procedure were the same as in Experiment 1 with the difference that this time, a white cross was added in the center of each image and participants were asked to perform their saccades toward the white cross in the center of the lateral image containing the target stimulus.

## Experiment 2

### Materials and Methods

#### Participants

Fourteen participants (eight females; mean age ± SD = 24.6 ± 4.9 years) with normal or corrected-to-normal vision, recruited from University Grenoble Alpes, took part in the experiment. They all came twice to complete two experimental sessions, one with faces as target stimuli and one with vehicles as target stimuli. All participants gave their informed written consent before participating in the study, which was carried out in accordance with the Code of Ethics of the World Medical Association (Declaration of Helsinki) for experiments involving humans and was approved by the ethic committee of University Grenoble Alpes.

#### Stimuli and Procedure

**Figure 4:**
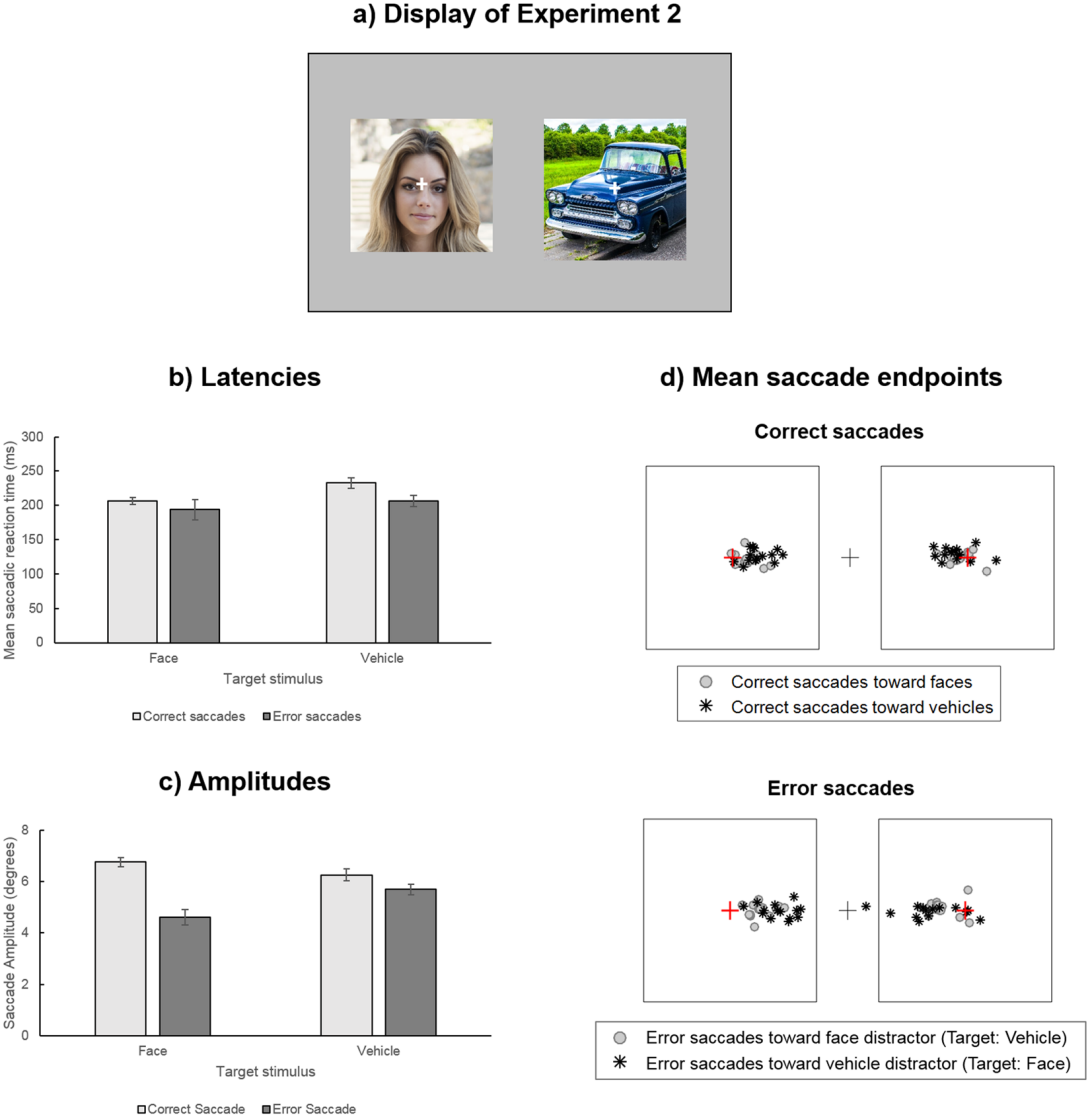
(**a**) Illustration of the display used in Experiment 2. Participants were instructed to perform their saccades toward the white cross in the center of the image containing the target category. (**b**) Mean saccadic reaction times (in milliseconds) and (**c**) mean amplitude (in degrees) of correct (light grey) and error saccades (dark grey), according to the target stimulus. Error bars indicate standard error. (**d**) Mean ending point of correct (top) and error saccades (bottom) of each participant. The black boxes represent the edges of lateral images, the central cross represents the fixation point and the red crosses represent the white cross added at the center of lateral images.

Stimuli and procedure were exactly the same as in Experiment 1. The right eye was recorded in 11 out of the 14 participants. The only difference was that a white fixation cross subtending 0.73° of visual angle was added in the center of each lateral image (Fig. 4a), displayed at 7.6° of eccentricity from the central fixation point, and participants were instructed to perform saccades as fast as possible toward the cross in the center of the image containing the target stimulus.

#### Data analyses

We extracted saccade parameters detected by the Eyelink software and did the same analyses as in Experiment 1. Trials where SRT was inferior to 50 ms or where a blink occurred during stimulus presentation were discarded from the analysis. This resulted in removing 1.21% of the trials.

### Results

#### Accuracy

We performed a paired t-test on mean error rates with Target Stimulus (Face, Vehicle) as a within-subject factor. Results revealed that participants made significantly more errors when the target stimulus was a vehicle than when it was a face (Vehicle as target: 26.59 ± 11.45%, Face as target: 11.29 ± 5.87%, t_13_ = 7.29, p < 0.001).

#### Latency and Amplitude of the first saccade

ANOVAs with Target Category (Face, Vehicle) and Saccade Accuracy (Correct, Error) as within-subject factors were performed on mean SRT of the first saccade (in ms) and saccade amplitude (in degrees).

The ANOVA performed on mean SRT (Fig. 4b) revealed a main effect of Target Category (F_1,23_ = 17.36, p < 0.005, η_p_^2^ = 0.572) and a main effect of saccade Accuracy (F_1,23_ = 7.76, p < 0.05, η_p_^2^ = 0.374). Participants initiated saccades faster when the target stimulus was a face (200 ± 37 ms) than when it was a vehicle (220 ± 30 ms) and they were slower to initiate correct (220 ± 24 ms) than error saccades (200 ± 43 ms). There was no interaction between the two factors (F_1,23_ = 1.68, p = 2.22, η_p_^2^ = 0.115).

The ANOVA performed on mean Saccade Amplitude (Fig. 4c) revealed a significant main effect of Target Category (F_1,23_ = 6.44, p < 0.05, η_p_^2^ = 0.331) and a main effect of Saccade Accuracy (F_1,23_ = 86.13, p < 0.0001, η_p_^2^ = 0.868). Saccades were overall larger when the target stimulus was a vehicle (5.98 ± 0.87°) than when it was a face (5.68 ± 1.42°) and error saccades (5.23 ± 0.96°) were shorter than correct saccades (6.51 ± 0.74°). Furthermore, there was a significant interaction between these two factors (F_1,23_ = 59.74, p < 0.0001, η_p_^2^ = 0.822). Pairwise comparisons showed that correct saccades were larger when the target stimulus was a face than a vehicle (face: 6.75 ± 0.65°, Vehicle: 6.26 ± 0.89°, p < 0.005) but this difference was reversed for error saccades which were larger when the target stimulus was a vehicle (i.e. face distractor: 5.70 ± 0.77°) than when it was a face (i.e. vehicle distractor: 4.61 ± 1.14°, p < 0.001), suggesting overall larger saccades directed toward faces (either as target or distractor) than toward vehicles. Finally, error saccades were shorter than correct saccades for both target categories (both ps < 0.005).

**Figure 5:**
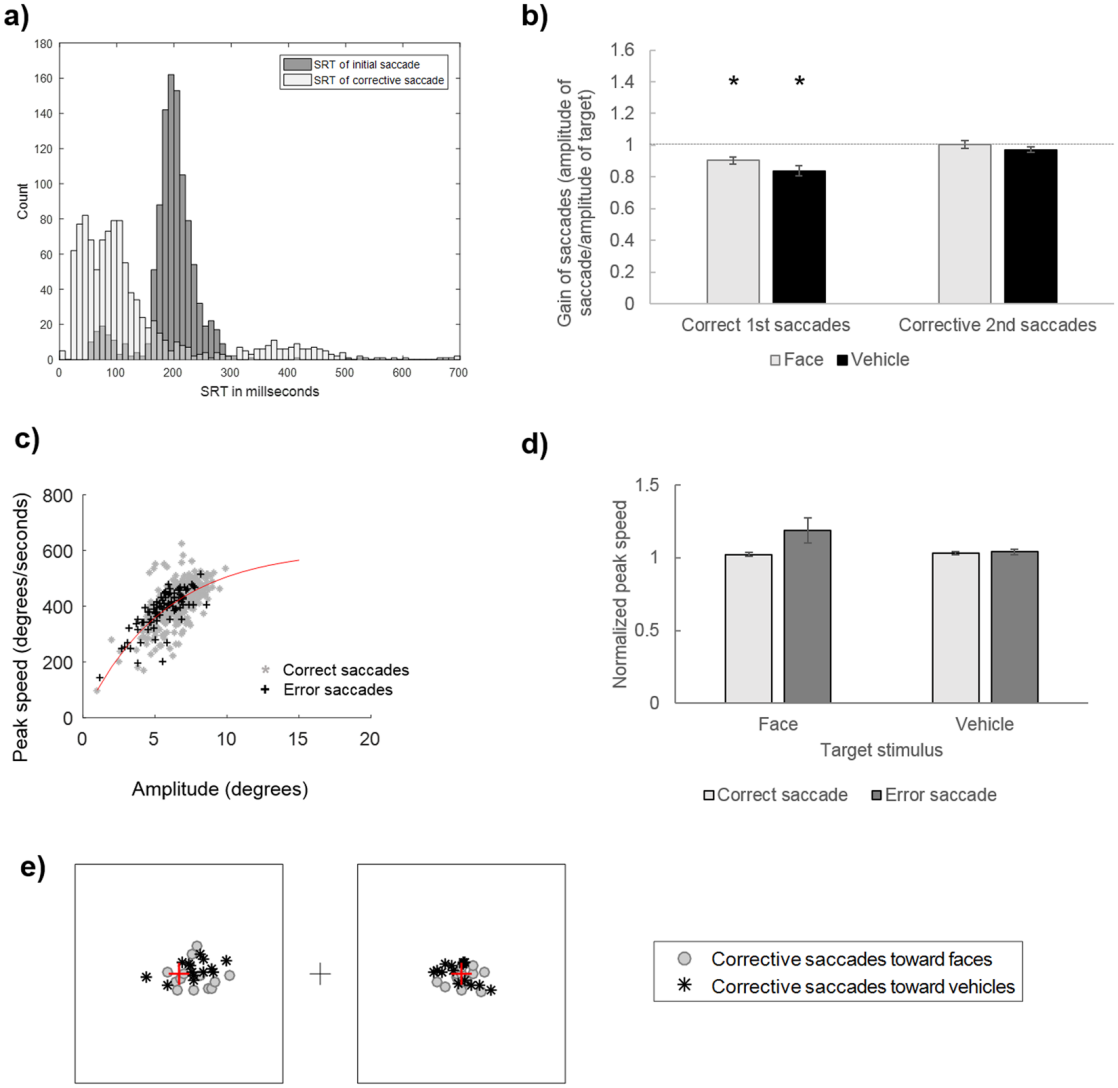
(**a**) Distributions of latencies of initial error saccades (dark grey) and of ensuing corrective saccades (light grey). (**b**) Mean gains of correct first saccades and second corrective saccades according to their target. The dotted line indicates a gain of 1, suggesting a perfect match between the amplitude of saccades and the amplitude of the target. * indicates significant difference from 1. (**c**) Main sequence of correct and error saccades of a participant. The red line corresponds to the exponential function fitted to the main sequence of correct saccades. **(d**) Mean normalized peak speed of correct and error saccades, according to the category of the target. Error bars indicate standard error. (**e**) Mean ending point of corrective saccades of each participant. The black boxes represent the edges of lateral images, the central cross represents the fixation point and the red crosses represent the white cross added at the center of lateral images.

It should be noted that the mean amplitude of correct saccades (6.75° for saccades toward faces, 6.26° for saccades toward vehicles) were below the amplitude of the target crosses added in the center of images (7.6°, see Fig. 4c,d). In order to test whether correct saccades significantly undershot the targets, we computed the mean gain of correct saccades (i.e. ratio of observed amplitude on the amplitude required to reach the target cross, based on the starting point of the saccade) for each participants and compared it against 1 (i.e. equivalence between the amplitude of the saccade and the eccentricity of the target) using one-sample t-tests. Results revealed that the gain of correct saccades toward faces and vehicles were significantly below 1, suggesting that they undershot the targets by 10 and 16% respectively (Face as target: mean gain ± SD: 0.90 ± 0.09, t_13_ = −4.13, p < 0.05; Vehicle as target: 0.84 ± 0.12, t_13_ = −5.24, p < 0.05, Fig. 5b,e).

#### Analysis of corrective saccades

As for Experiment 1, we examined whether error saccades (representing 18.2% of the trials) were followed by a corrective one. A second saccade was considered as corrective if its ending point was on the side of the display where the target was present (i.e. if they crossed the central fixation point). This represented 88.81% of the saccades following an error. Again, these corrective saccades had very short latencies with a median of 96 ms (min = 21 ms; max = 1010 ms; mean = 147 ms, SD = 140 ms), suggesting that more than half of corrective saccades had latencies below 100 ms (see Fig. 5a). In order to test whether corrective saccades differed according to their target, we performed paired t-tests comparing the proportion, amplitude and latency of corrective saccades for Face and Vehicle. However, as in Experiment 1, there was no significant difference between the proportion (t_13_ = −0.19, p = 0.85), latency (t_13_ = 1.42, p = 0.18) or amplitude (t_13_ = 0.89, p = 0.39) of corrective saccades according to their target stimulus.

We also examined whether saccades following an error compensated for the amplitude of the error by first examining the correlation between the mean amplitudes of the error and corrective saccades. As in Experiment 1, we observed significant correlations between the mean amplitudes of initial and second saccades for both target conditions (Face as target: r = 0.70, p < 0.01; Vehicle as target: r = 0.70, p < 0.01), indicating that the higher the amplitude of error, the higher the amplitude of the second saccade toward the target. Furthermore, as this experiment involved a specific location to reach, we also compared the amplitude of corrective saccades with the amplitude that would have been required to reach the target (i.e. distance to the target after an error saccade). This was done by computing the gain of corrective saccades. A gain of 1 indicates a perfect precision of saccades, whereas gains inferior/superior to 1 indicate an under/overshooting of the target, respectively. Thus, if the corrective saccade did not accurately compensate for the amplitude of the error, its gain should be inferior to 1 and to the gain of correct saccades. One-sample t-tests comparing the gain of corrective saccades against 1 revealed no significant difference (Face as target: mean gain ± SD: 1.00 ± 0.09, t_13_ = 0.20, p > 0.99; Vehicle as target: 0.97 ± 0.07, t_13_ = −1.59, p = 0.53), Critically, paired t-tests further showed that the gain of corrective saccades was higher than that of correct saccades for both target conditions (Face as target: t_13_ = 3.02, p < 0.05; Vehicle as target: t_13_ = 4.73, p < 0.05), indicating that corrective saccades were more accurate than initially correct saccades in terms of spatial precision.

#### Analysis of saccade kinematics

As for Experiment 1, we tested whether hypometric error saccades also deviated from correct saccades in terms of their main sequence, by examining normalized peak speed of saccades (see Fig. 5c and Supplementary Fig. S3). Here again, results revealed a main effect of Saccade Accuracy (F_1,13_ = 5.07, p < 0.05, η_p_^2^ = 0.280), suggesting a violation of the main sequence for error saccades: normalized peak speed of error saccades (1.11 ± 0.16) were higher than that of correct saccades (1.03 ± 0.04). There was no main effect of Target Category (F_1,13_ = 2.38, p = 0.15 η_p_^2^ = 0.155), and no interaction between this factor and Saccade Accuracy (F_1,13_ = 3.63, p = 0.08, η_p_^2^ < 0.218).

### Discussion

Results of Experiment 2 replicated the main findings of Experiment 1, indicating that (1) saccades toward vehicles are shorter than saccades toward faces and (2) error saccades are shorter than correct saccades. Critically, these effects persisted even though participants were explicitly asked to perform their saccade toward a specific location on the target image, suggesting they reflect an influence of target/distractor content and response accuracy on saccade programming that is beyond voluntary control. As in Experiment 1, we also observed that hypometric error saccades deviated from the main sequence of correct saccades, suggesting their interruption by concurrently programmed saccades. Critically, although initially correct saccades toward faces and vehicle both undershot the target, corrective saccades following an error did reach it with high precision. Furthermore, hypometria observed for correct saccades toward vehicles, relative to faces was not observed for corrective saccades. These results therefore suggest that saccades following an error were programmed to compensate for the amplitude of error and were no longer influenced by the content of the target/distractor images. Overall, results of Experiment 2 support a strong influence of face stimuli on saccade programming but also suggest that online correction of error saccades can occur during the saccadic choice task.

It can be noted that in comparison to Experiment 1, latencies of saccades in Experiment 2 were slightly longer, with an increase of ~20 ms in all experimental conditions. As the only difference between Experiment 1 and 2 was that Experiment 2 required the programming of the saccade endpoint in addition to saccade direction, the mean difference between saccade latencies in the two experiments therefore suggests that the programming of saccade endpoint, relative to the programming of its direction only, comes with a processing cost that takes about 20 ms. This cost is comparable to what has been observed in previous studies^44,45^. For example, using an anti-saccade task, Evdokimidis *et al*.^44^ found that when participants were asked to perform anti-saccades in a mirror position relative to the target, latencies were 19 ms longer than when they were simply asked to perform anti-saccade in the opposite direction of the target. The present data therefore support the view that programming of saccade amplitude and direction may rely on different processes.

## General Discussion

The present study investigated how the content of simultaneously displayed visual stimuli can influence saccade programming during a saccadic choice task, with a particular focus on face stimuli. Our study replicated previous findings indicating that, relative to vehicle stimuli, face stimuli elicit faster saccades, but also more involuntary error saccades when they are defined as distractors^3,4,12–14,18^. Critically, the examination of saccade amplitude during this task additionally revealed two new main findings. First, we observed that amplitude of saccades was modulated by the content of the target/distractor stimuli: Saccades toward vehicles (faces as distractor) were shorter than saccades toward faces (vehicle as distractor). Second, we observed that error saccades were shorter than correct saccades suggesting their interruption to initiate a corrective saccade. In the following, we discuss the significance of these results and their potential underlying neural mechanisms, as well as their implication for studies on eye movements.

### Saccade amplitude is modulated by the content of the stimuli

Throughout two experiments, we consistently observed that saccades toward faces were larger than saccades toward vehicles. This effect persisted even if participants were explicitly required to perform their saccades toward a specific location within the images (Experiment 2) indicating it could not be voluntarily controlled. Results of Experiment 2 revealed that all saccades actually tended to undershoot the target point, but to a greater extent when they were directed toward vehicle than face stimuli, suggesting that saccades toward vehicles were rather hypometric relative to saccades toward faces. These results therefore indicate that the content of visual stimuli influences the programming of saccade amplitude in a saccadic choice task.

Previous studies proposed that when a task involves multiple potential targets (e.g., as in saccadic choice task or visual search task) or the potential execution of eye movements toward different locations (e.g., as in the anti-saccade task), saccade programs oriented toward these different goals could be developed in parallel and compete with each other through mutual inhibition on a common saccade map representing the location of the saccadic goals^30,34,36–39,46,47^. It is generally agreed that such a map would be represented in intermediate layers of the superior colliculus (iSC) which integrate external signals (e.g., visual inputs from occipital visual areas) and internal/goal-related signals (e.g., related to task instructions, via inputs from the frontal eye fields and dorsolateral prefrontal cortex) to select among possible saccadic goals^30,34,36,48–52^. The saccade program developing the fastest (i.e. reaching a threshold for activation first) would win the competition and be executed while the concurrent program would be cancelled. However, if a competing program develops fast enough, it might still influence and interfere with the programming and execution of the winning program. This would arise through increased activity of neurons coding for the alternative saccade goal, thereby weakening activity of neurons coding for the goal of the winning program^49^, resulting in the reduced amplitude of the executed saccade^30,46^. Within this framework, the general undershoot observed for correct saccades could be interpreted as resulting from the interfering influence of a competing saccade program directed toward the distractor image.

In our saccadic choice task, the face and vehicle images were displayed simultaneously on the left and right visual fields, thus involving the generation of two potential saccade programs, toward the left and right crosses, interfering with each other. Critically, the fact that saccades toward vehicles were even shorter than saccades toward faces suggests that (1) the content of the images played a critical role in these competitive interactions and (2) the saccade program toward a face stimulus interfered more strongly (through greater weight/stronger inhibition) with the saccade program toward a vehicle stimulus than the other way around. A greater weight of saccade programs toward face stimuli would also be consistent with the higher error rate of saccadic responses toward face than vehicle distractors observed in both experiments, suggesting that the saccade program toward faces actually won the competition in a substantial amount of trials. This is also in line with previous studies showing that eye movements toward faces cannot be easily inhibited^4,18,19^. Overall, our results therefore suggest for the first time that the characteristics of visual stimuli, such as their behavioral relevance can come into play in the interactions underlying the parallel programming of saccades, by modulating the relative weight of competing programs for saccade target selection.

Recent studies suggested that this bias for face stimuli could be mediated by rapid processing of visual information via a subcortical retino-tectal pathway, through which part of visual information exiting ganglion retinal cells directly projects to the superficial layers of the superior colliculus (sSC) before reaching the pulvinar and the amygdala^53–56^. Nakano *et al*.^53^ for example argued that the speed of processing along in the main retino-geniculo-cortical pathway (the earliest visual response latencies observed in human visual cortical areas are of 56 ms in V1, and 70–80 ms in V3 and V4^57^ cited in^53^, together with the time required to generate a saccade (20–30 ms) is too slow to account for the fast saccadic responses toward faces in just 100–110 ms, as previously observed by Crouzet *et al*.^3^ However, electrophysiological recordings in monkeys^54,56^ showed that neurons in the sSC and in the pulvinar exhibit distinct response to face-like stimuli within the first 25 ms and 50 ms following stimulus onset, respectively. As the pulvinar directly projects to the lateral intraparietal cortex, involved in saccade generation with latencies of 30 ms, such timings would be more compatible with the speed of face processing previously observed. Interestingly, the retino-tectal pathway is thought to be particularly involved in the detection of behaviorally-relevant stimuli such as faces See for a review^55^. However, exactly how fast detection of faces via the sSC in turn modulates the weight of a saccade program toward this stimulus in the iSC remains to be addressed.

Another remaining question pertains to the nature of visual information rapidly extracted from face stimuli which could underlie these effects. In a recent study, Guyader *et al*.^14^ examined the role of low-level properties of stimuli such as color and spatial frequencies for fast detection of faces during a saccadic choice task. In this study, stimuli were either colored, gray-scaled, filtered in low spatial frequencies or in high spatial frequencies. They observed faster saccadic reaction times (SRTs) toward face than other target categories in all viewing conditions, but SRTs toward face targets were faster when the images were unfiltered (colored and grey-scaled) and filtered in LSF than when they were filtered in HSF. These results suggested that rapid detection of faces could be partly mediated by fast processing of their LSF content. However, previous research supports the idea that the bias for faces during saccade programming cannot be totally accounted for by low-level properties of visual stimuli. For example, using an anti-saccade task, a couple of studies showed that participants made more errors and were slower when they had to perform saccades in the direction opposite to upright faces than to inverted faces^18^ or phase-scrambled versions of faces^4^ sharing the same low-level properties. These results therefore additionally suggest that the orienting bias toward faces rather reflects an influence of high-level processing on saccade programming. Further studies manipulating both low- and high-level properties of stimuli during a saccadic choice task would be needed to disambiguate that question.

It should be noted that we cannot totally rule out that the difference in saccade amplitude toward faces and vehicles simply results from a general tendency to make larger saccades toward faces than vehicles, irrespective of the presence of a distractor or a concurrent saccade program. Indeed, results of a control experiment (see Supplementary Data) in which participants had to perform a saccade toward a central cross in a face or vehicle image displayed without a distractor suggested that saccades toward faces are still slightly but significantly larger than saccades toward vehicles, albeit to a lesser degree than what was observed in our Experiments. This therefore further supports the idea of an influence of stimulus processing on saccade programming. Interestingly, this bias appeared to be abolished under particular circumstances as we observed that corrective saccades following an error could reach the target, with no significant difference according to the content of the target stimulus. This indicates that participants *were* in some cases able to perform spatially accurate saccades toward the target point, irrespective of the image contents. Furthermore, this suggests that after an error saccade toward the distractor was inhibited, saccade toward target were no longer interfered and could reach the target point with high precision (see also our discussion below).

### Error saccades are corrected on-line

In comparison to previous studies using a saccadic choice task which mainly focused on the analysis of correct saccades, the present study further examined the characteristics of error saccades. First, we observed in both experiments that error saccades were hypometric relative to correct saccades. This finding echoes previous reports of hypometric erroneous saccades in tasks involving saccadic responses such as the visual search task^30,33,34,41^ or the anti-saccade task^31,46,47^. Second, these error saccades were followed in the large majority of trials by a second corrective saccade bringing the gaze to the target location after a very short delay (median < 100 ms), well below the range of saccade latencies observed for the first correct and error saccades (median around 200 ms). Such extremely short inter-saccadic intervals were previously reported in studies involving the execution of two consecutive saccades and were taken as evidence that the programming of the second saccade occurred in parallel to the execution of the initial one^30,31,34,35,38,58^. At the neurobiological level, this would translate into a rise in activity of neurons in the iSC and frontal eye fields coding for the second saccade goal, while (or even before) the first saccade is being executed^36,52,59,60^.

Finally, the examination of saccade kinematics revealed that, on average, error saccades deviated from the main sequence of correct saccades, with higher peak velocities than what was predicted from the observed amplitude. Overall, these results suggest that error saccades toward a distractor image were interrupted while they were being executed, to initiate a concurrently-programmed corrective saccade toward the target. Only a few studies have reported mid-flight interruption of saccades, in which the brief appearance of a distractor (e.g., a flash) resulted in hypometric saccades toward a target, with abnormal speed profiles given their amplitude^43,61^. However, the interruption of an erroneous saccadic response to initiate a corrective saccade in a different direction has, to our knowledge, not been reported. For example, using a visual search task, McPeek *et al*.^30^ observed that, although error saccades were hypometric and followed by concurrently programmed corrective saccades, they did not deviate from the main sequence and were interpreted as reflecting diminished neural activity corresponding to the first saccade goal, due to the competing saccade program toward the second goal. It should however be noted that in most studies reporting hypometric error saccades associated with short-latency corrective saccade, kinematics of error saccades were not examined e.g.^31,31,34,41,46,47^. Thus, whether mid-flight interruption of error saccades occurred in these studies could not be addressed. Interestingly, the fact that error saccades toward vehicle distractors were even shorter than error saccades toward face distractors suggests their interruption occurred earlier when a concurrent saccade was programmed toward a face than toward a vehicle stimulus, respectively. In that sense, results on error saccades support the above-mentioned assumption of a greater weight for saccade programs directed toward faces to interrupt a saccade program toward another stimulus.

Finally, the examination of concurrently-programmed saccades following an error revealed that they compensated for the amplitude of the initial error saccade and reached the target location, irrespective of the content of stimuli. This contrasts with the relatively lower precision of correct saccades, which were found to systematically undershoot the target and to a higher degree when it was a vehicle. We previously proposed that undershoot of correct saccades could be explained by the influence of the distractor image on the programming of saccade toward the target. In this context, the higher gain of corrective saccades suggests that after the interruption of an error saccade program, this program did no longer influence the amplitude of the concurrently-programmed corrective saccade toward the target. It is generally agreed that sensitivity to visual information is reduced and non-motion information suppressed while a saccade is being performed^62–64^. Therefore, the relative distance to the target could not be computed based on visual information during the execution of the initial error saccade. Furthermore, the latencies of corrective saccades were too short to allow (1) the computation of the target’s distance based on the new eye position and (2) the programming of a saccade toward it. Therefore, it can be assumed that the target location, relative to the eye position, was computed before the initiation of the first error saccade (i.e. while fixating on the central cross) and was updated at the end of the error saccade. This proposal is supported by neurophysiological and behavioral studies showing that the oculomotor system generates predictions based on pre-saccadic information, in order to facilitate processing of post-saccadic information^30,34,65–67^.

Overall, the data obtained in the present study point to the ability for the oculomotor system to efficiently perform an online correction of saccadic response, through the development of a saccade program while a first erroneous saccade is being executed, resulting in its interruption, and then the execution of an accurate saccade toward the target. It should be noted that the notion of ‘correction’ does not necessarily imply that participants were aware of an error and voluntarily corrected for it. On the contrary, a couple of studies indicated that hypometric error saccades followed by short-latency corrective saccades were more likely to be observed when participants were unaware of the initial error^47,68^.

## Conclusion

The present study provides new insights on the interactions between the oculomotor system and the properties of visual stimuli, by showing for the first time that the programming of saccades during a saccadic choice task is influenced by the content of the saccadic goal(s). These findings further support previous studies showing that faces constitute particular stimuli for the visual system and elicit characteristic behavioral responses, relative to other categories of visual stimuli. Additionally, analysis of saccade amplitude and saccade kinematics in the present study allowed us to further precise the processes at hand during the saccadic choice task, by showing that efficient online correction of error saccades can occur at least during this task. Overall, these findings emphasize the importance of using ecological and behaviorally-relevant stimuli but also the relevance of examining oculomotor parameters such as saccade amplitude to better characterize and understand the functioning of the visual and oculomotor systems.

## Electronic supplementary material

Supplementary Information

## References

[1] Liu J, Harris A, Kanwisher N (2002). Stages of processing in face perception: an MEG study. Nat. Neurosci..

[2] Liu J, Higuchi M, Marantz A, Kanwisher N (2000). The selectivity of the occipitotemporal M170 for faces. Neuroreport.

[3] Crouzet SM, Kirchner H, Thorpe SJ (2010). Fast saccades toward faces: face detection in just 100 ms. J. Vis..

[4] Morand SM, Grosbras MH, Caldara R, Harvey M (2010). Looking away from faces: influence of high-level visual processes on saccade programming. J Vis.

[5] Haxby JV, Hoffman EA, Gobbini MI (2000). The distributed human neural system for face perception (Record Supplied By Publisher). Trends Cogn Sci.

[6] Farah MJ, Wilson KD, Drain M, Tanaka JN (1998). What is ‘Special’ about Face Perception?. Psychol. Rev..

[7] Foulsham T, Cheng JT, Tracy JL, Henrich J, Kingstone A (2010). Gaze allocation in a dynamic situation: Effects of social status and speaking. Cognition.

[8] Hirvenkari, L. *et al*. Influence of Turn-Taking in a Two-Person Conversation on the Gaze of a Viewer. *PLoS One* **8**, (2013). 10.1371/journal.pone.0071569 PMC3741179 23951192

[9] Coutrot A, Guyader N (2014). How saliency, faces, and sound influence gaze in dynamic social scenes. J. Vis..

[10] Tilke, J., Ehinger, K., Durand, F. & Torralba, A. Learning to predict where humans look. *Proc. IEEE Int. Conf. Comput. Vis*. 2106–2113, 10.1109/ICCV.2009.5459462 (2009).

[11] Marat S, Rahman A, Pellerin D, Guyader N, Houzet D (2013). Improving Visual Saliency by Adding ‘Face Feature Map’ and ‘Center Bias’. Cognit. Comput..

[12] Crouzet, S. M. & Thorpe, S. J. Low-level cues and ultra-fast face detection. *Front. Psychol*. **2** (2011). 10.3389/fpsyg.2011.00342 PMC3221302 22125544

[13] Boucart M (2016). Finding faces, animals, and vehicles in far peripheral vision. J. Vis..

[14] Guyader, N., Chauvin, A., Boucart, M. & Peyrin, C. Do low spatial frequencies explain the extremely fast saccades towards human faces? *Vision Res*., 10.1016/j.visres.2016.12.019 (2017). 10.1016/j.visres.2016.12.019 28202396

[15] Kirchner H, Thorpe SJ (2006). Ultra-rapid object detection with saccadic eye movements: Visual processing speed revisited. Vision Res..

[16] Fischer B, Weber H (1993). Express saccades and visual attention. Behav. Brain Sci..

[17] Kalesnykas RP, Hallett PE (1987). The differentiation of visually guided and anticipatory saccades in gap and overlap paradigms. Exp. Brain Res..

[18] Gilchrist ID, Proske H (2006). Anti-saccades away from faces: Evidence for an influence of high-level visual processes on saccade programming. Exp. Brain Res..

[19] Bindemann M, Burton AM, Langton SRH, Schweinberger SR, Doherty MJ (2007). The control of attention to faces. J. Vis..

[20] Quaia C, Lefèvre P, Optican LM (1999). Model of the control of saccades by superior colliculus and cerebellum. J. Neurophysiol..

[21] Kapoula Z, Robinson DA (1986). Saccadic undershoot is not inevitable: Saccades can be accurate. Vision Res..

[22] Kowler E, Anderson E, Dosher B, Blaser E (1995). The role of attention in the programming of saccades. Vision Res..

[23] Collewijn H, Erkelens C, Steinman R (1988). Binocular co-ordination fo human horisontal saccadic eye movements. J. Physiol..

[24] Coren S, Hoenig P (1972). Effect of non-target stimuli upon length of voluntary saccades. Percept. Mot. Ski..

[25] Van der Stigchel S, Nijboer TC (2011). The global effect: what determines where the eyes land?. J. Eye Mov. Res..

[26] Vitu F (2008). About the global effect and the critical role of retinal eccentricity: Implications for eye movements in reading. J. Eye Mov. Res..

[27] Findlay JM (1982). Global visual processing for saccadic eye movements. Vision Res..

[28] Chou I (1999). han, Sommer, M. A. & Schiller, P. H. Express averaging saccades in monkeys. Vision Res..

[29] Findlay JM, Blythe HI (2009). Saccade target selection: Do distractors affect saccade accuracy?. Vision Res..

[30] McPeek RM, Skavenski AA, Nakayama K (2000). Concurrent processing of saccades in visual search. Vision Res..

[31] Weber H, Dürr N, Fisher B (1998). Effects of pre-cues on voluntary and reflexive saccade generation. I. Anti-cues for pro-saccades. Exp. Brain Res..

[32] Godijn R, Theeuwes J (2002). Oculomotor capture and Inhibition of Return: Evidence for an oculomotor suppression account of IOR. Psychol. Res..

[33] Findlay JM, Brown V, Gilchrist ID (2001). Saccade target selection in visual search: The effect of information from the previous fixation. Vision Res..

[34] Godijn R, Theeuwes J (2002). Programming of Endogenous and Exogenous Saccades: Evidence for a Competitive Integration Model. J. Exp. Psychol. Hum. Percept. Perform..

[35] Becker W, Jürgens R (1979). An analysis of the saccadic system by means of double step stimuli. Vision Res..

[36] McPeek RM, Keller EL (2002). Superior Colliculus Activity Related to Concurrent Processing of Saccade Goals in a Visual Search Task. J. Neurophysiol..

[37] Findlay, J. M. & Walker, R. A model of saccade generation based on parallel processing and competitive inhibition. *Behav. Brain Sci*. **22**, 661–74; discussion 674–721 (1999). 10.1017/s0140525x99002150 11301526

[38] Walker R, McSorley E (2006). The parallel programming of voluntary and reflexive saccades. Vision Res..

[39] Ramakrishnan A, Chokhandre S, Murthy A (2010). Voluntary Control of Multisaccade Gaze Shifts During Movement Preparation and Execution. J. Neurophysiol..

[40] Ionescu, G., Guyader, N. & Guérin-dugué, A. SoftEye software. *IDDN. FR* **1** (2009).

[41] Viviani P, Swensson RG (1982). Saccadic eye movements to peripherally discriminated visual targets. J. Exp. Psychol. Hum. Percept. Perform..

[42] Bahill AT, Clark MR, Stark L (1975). The Main Sequence, A Tool for Studying Human Eye Movements. Math. Biosci..

[43] Buonocore A, McIntosh RD, Melcher D (2016). Beyond the point of no return: effects of visual distractors on saccade amplitude and velocity. J. Neurophysiol..

[44] Evdokimidis I, Tsekou H, Smyrnis N (2006). The mirror antisaccade task: direction-amplitude interaction and spatial accuracy characteristics. Exp. Brain Res..

[45] Allik J, Toom M, Luuk A (2003). Planning of saccadic eye movements. Psychol Res.

[46] Massen C (2004). Parallel programming of exogenous and endogenous components in the antisaccade task. Q. J. Exp. Psychol. Sect. A Hum. Exp. Psychol..

[47] Mokler A, Fischer B (1999). The recognition of errors and corrections in an antisaccade task. Exp. Brain Res..

[48] Trappenberg TP, Dorris MC, Munoz DP, Klein RM (2001). A Model of Saccade Initiation Based on the Competitive Integration of Exogenous and Endogenous Signals in the Superior Colliculus. J. Cogn. Neurosci..

[49] Dorris MC, Olivier E, Munoz DP (2007). Competitive Integration of Visual and Preparatory Signals in the Superior Colliculus during Saccadic Programming. J. Neurosci..

[50] Meeter M, Van Der Stigchel S, Theeuwes J (2010). A competitive integration model of exogenous and endogenous eye movements. Biol. Cybern..

[51] Lee C, Rohrer WH, Sparks DL (1988). Population coding of saccadic eye movements by neurons in the superior colliculus. Nature.

[52] Munoz, D. P. & Schall, J. D. Concurrent, Distributed Control of saccade initiation in the frontal eye field and superior colliculus. *Super. Colliculus New approaches stydying sensorimotor Integr*. 55–82, 10.1201/9780203501504 (2004).

[53] Nakano T, Higashida N, Kitazawa S (2013). Facilitation of face recognition through the retino-tectal pathway. Neuropsychologia.

[54] Nguyen MN (2014). Neuronal responses to face-like and facial stimuli in the monkey superior colliculus. Front. Behav. Neurosci..

[55] Soares SC, Maior RS, Isbell LA, Tomaz C, Nishijo H (2017). Fast detector/first responder: Interactions between the superior colliculus-pulvinar pathway and stimuli relevant to primates. Front. Neurosci..

[56] Nguyen MN (2013). Neuronal responses to face-like stimuli in the monkey pulvinar. Eur. J. Neurosci..

[57] Yoshor D, Bosking WH, Ghose GM, Maunsell JHR (2007). Receptive fields in human visual cortex mapped with surface electrodes. Cereb. Cortex.

[58] McSorley E, McCloy R, Williams L (2016). The concurrent programming of saccades. PLoS One.

[59] Sharika KM, Ramakrishnan A, Murthy A (2008). Control of Predictive Error Correction During a Saccadic Double-Step Task. J. Neurophysiol..

[60] Murthy A (2006). Frontal Eye Field Contributions to Rapid Corrective Saccades. J. Neurophysiol..

[61] Edelman JA, Xu KZ (2009). Inhibition of Voluntary Saccadic Eye Movement Commands by Abrupt Visual Onsets. J. Neurophysiol..

[62] Castet, E. & Masson, G. S. Motion perception during saccadic eye movements. *Nat. Neurosci*. 10.1038/72124(2000). 10.1038/72124 10649574

[63] Krekelberg B (2010). Quick guide Saccadic suppression. Curr. Biol..

[64] Castet E, Jeanjean S, Masson GS (2002). Motion perception of saccade-induced retinal translation. Proc. Natl. Acad. Sci..

[65] Fabius JH, Fracasso A, Van Der Stigchel S (2016). Spatiotopic updating facilitates perception immediately after saccades. Sci. Rep..

[66] Van der Stigchel S, Hollingworth A (2018). Visuospatial Working Memory as a Fundamental Component of the Eye Movement System. Curr. Dir. Psychol. Sci..

[67] Duhamel J, Colby CL, Goldberg ME (1992). The Updating of the Representation of Visual representation. Science (80-.)..

[68] Nieuwenhuis S, Richard Ridderinkhof K, Blom J, Band GPH, Kok A (2001). Error-related brain potentials are differentially related to awareness of response errors: Evidence from an antisaccade task. Psychophysiology.

