# Supplementary material for: Face perception influences the programming of eye movements
Source: Sci Rep. 2019 Jan 24;9:560. doi: 10.1038/s41598-018-36510-0 (PMC6346063; doi:10.1038/s41598-018-36510-0)
Supplement: Supplementary file 1 — Supplementary Information [file 41598_2018_36510_MOESM1_ESM.pdf]

Face perception influences the programming of eye movements

Louise Kauffmann[1,2], Carole Peyrin[2], Alan Chauvin[2], Léa Entzmann[1], Camille Breuil[1] & Nathalie Guyader[1]

[1] Univ. Grenoble Alpes, CNRS, Grenoble INP, GIPSA-lab, 38000 Grenoble, France
[2] Univ. Grenoble Alpes, CNRS, LPNC, 38000 Grenoble, France

Supplementary Information

Supplementary Analysis S1:

In our experiment, there were more images of face than vehicles for which the main object was centered on the symmetrical midline. In order to test whether the larger amplitude of saccades toward faces relative to vehicles targets in our experiments could be explained by the larger proportion of symmetry around the vertical midline for face than vehicle stimuli, we re-analyzed the data of both experiments by only taking into account trials in which such images were used as target. In order to equalize our samples, we randomly selected 24 images of symmetrical faces to match the 24 images of symmetrical vehicles.

For both experiments, we found the same pattern of results as those reported in the manuscript: correct saccades toward face targets had a larger amplitude than correct saccade toward vehicle targets (see Supplementary Table S1 below).

Supplementary Table S1: Results of the statistical analysis comparing the amplitude of saccades for trials in which only targets symmetrical around the vertical midline were taken into account (24 vehicle images and 24 face images). *SD: standard deviation, t: paired-sample t-test value, p = probability associated with the t value under to null hypothesis.*

	**Face target**		**Vehicle target**		**t**	***p***
	Mean	*SD*	Mean	*SD*		
**Experiment 1**	6.52	*0.77*	6.00	*0.59*	3.68	***0.002****
**Experiment 2**	6.65	*0.78*	6.17	*0.94*	2.87	***0.013****

The fact that these results remained even though stimuli were equalized in terms of symmetry around the vertical midline suggests that this factor did not account for the differences in saccade amplitudes toward face and vehicle stimuli.

Supplementary Analysis S2:

Saccades with latencies inferior to 50 ms were removed from the analysis in order to avoid including express saccades not related to the processing of stimuli. However, we cannot totally exclude that some saccades with extremely short latency included in our analyses were actually express saccades triggered by the offset of the fixation point. In order to address this question, we have plotted for Experiment 1 and Experiment 2 the amplitude (in degrees) as a function of the saccadic reaction time (or latency, in ms) of all analyzed saccades of all participants according to (1) the accuracy of the saccadic response (correct and error saccades) and (2) the category of the target stimulus (Face and Vehicle, see Supplementary Figure S1 below).

As can be seen on this figure, saccade amplitudes ranged between 0 and 11 degrees of visual angle, and latencies ranged between 50 and 700 ms. Although error saccades indeed exhibited on average shorter latencies than correct saccades, their distribution overlaps with the distribution of correct saccades both in terms of amplitude and latency. Interestingly, visual inspection of these graphs indicates that for each experimental condition, there is a small proportion of saccades exhibiting very short latencies (<100-120 ms) and amplitudes (< 2°; see bottom-left part of each graph) that are clearly separated from the main cluster of data. These particular data points are likely to correspond to express saccades triggered by the offset of the central fixation cross, which might therefore not reflect the processing of the images.

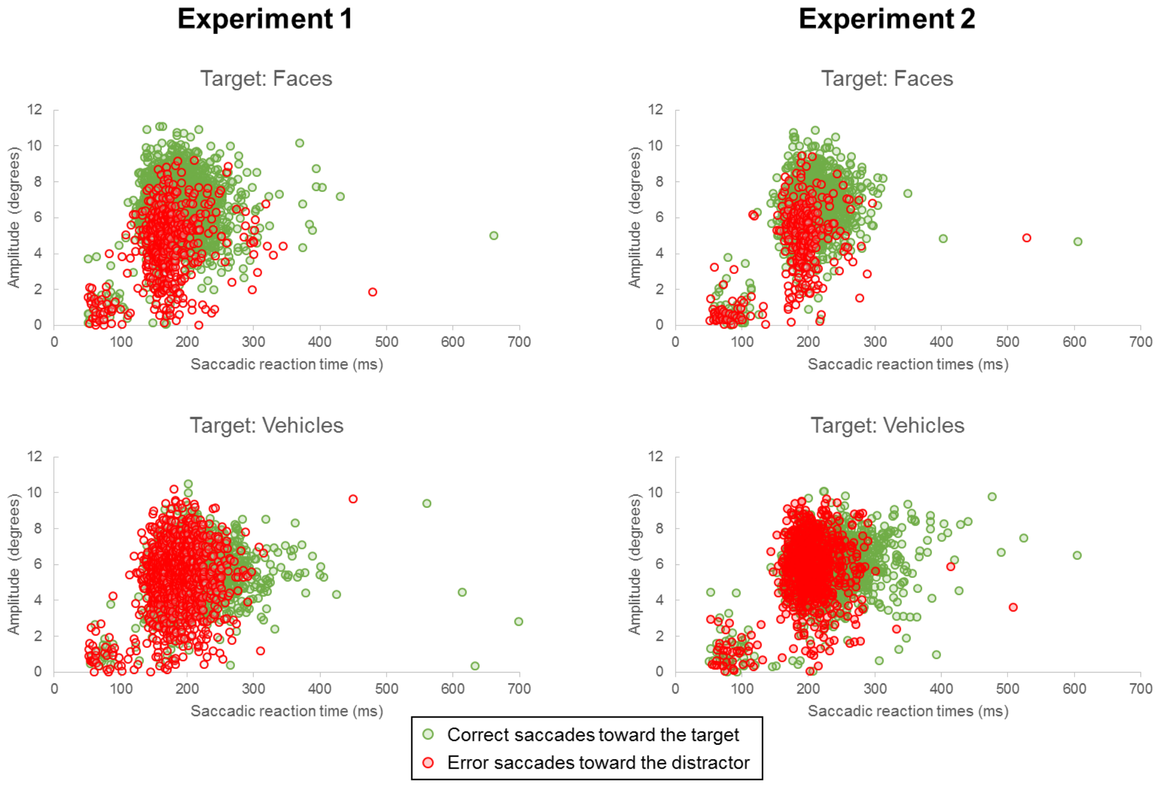

Supplementary Figure S1: Scatterplot of amplitude and saccadic reaction times of individual correct (in green) and error (in red) saccades, according to the category of the target stimulus.

In order to estimate the proportion of these express saccades, we divided the data into "express" and "stimulus-related" saccades and computed their number for each condition.

Based on visual inspection of these graphs, saccades were classified as “express” if they had a latency falling below 120 ms (which appears to be the latency at which the small cluster of suspected “express” saccades clearly separates from the main cluster of data). We found that the number of these express saccades was very low and relatively consistent across conditions (see Supplementary Table S2 below). We then calculated and compared the 95% confidence intervals for the proportion of Express and Stimulus-related correct and error saccades, according to the category of the target stimulus, using adjusted Wald intervals (Agresti & Caffo, 2000). Proportions of saccades were considered to be significantly different if the intervals did not overlap. As can be seen in Supplementary Table S2 below, the proportion of error (correct) “express” saccades did not significantly differ according to the category of the target stimulus, whereas the proportion of error (correct) saccades classified as “stimulus-related” was significantly higher (lower) when the target was a vehicle than when it was a face.

Supplementary Table S2: Estimated number and 95% confidence intervals (CI) for the proportion of “express” and “stimulus-related” saccades in each condition.

	**Target: Face**				**Target: Vehicle**			
	**Correct**		**Error**		**Correct**		**Error**	
	Number	*95% CI*	Number	*95% CI*	Number	*95% CI*	Number	*95% CI*
**Experiment 1**								
"Express" saccades	58	*[0.4752; 0.5683]*	53	*[0.4317; 0.5248]*	62	*[0.4921; 0.5835]*	53	*[0.4165; 0.5079]*
Stimulus-related" saccades	4189	*[0.8831; 0.8923]*	528	*[0.1077; 0.1169]*	3704	*[0.7797; 0.7916]*	1009	*[0.2084; 0.2203]*
**Experiment 2**								
"Express" saccades	73	*[0.5251; 0.6113]*	55	*[0.3887; 0.4749]*	57	*[0.4796; 0.5740]*	51	*[0.4260; 0.5204]*
Stimulus-related" saccades	2799	*[0.8968; 0.9074]*	302	*[0.0926; 0.1032]*	2321	*[0.7351; 0.7507]*	802	*[0.2493; 0.2649]*

Finally, we also estimated the impact of saccades classified as “express” on the main results reported in the manuscript by computing the mean latency and amplitudes of saccades classified as “stimulus-related”, excluding “express” saccades. We found that saccades classified as “express” had a very limited impact on the reported means, as they mainly resulted in decreasing the mean latencies and amplitudes by respectively 3-5 ms and 0.1-0.2 degrees, consistently across conditions (See Supplementary Table S3 below). Statistical analyses of data excluding these “express” saccades revealed the same pattern of results as reported in the manuscript for both experiments:

In terms of saccadic reaction time, participants initiated saccades faster when the target was a face than a vehicle (Experiment 1: $F_{1, 23} = 16.52$, $p < 0.001$; Experiment 2: $F_{1, 13} = 11.73$, $p < 0.005$) and they were slower to initiate error than correct saccades (Experiment 1: $F_{1, 23} = 29.33$, $p < 0.001$; Experiment 2: $F_{1, 13} = 4.71$, $p < 0.05$). Furthermore, the difference in latencies according to the target stimulus was more pronounced for correct than error saccades (Experiment 1: $F_{1, 23} = 13.19$, $p < 0.005$; Experiment 2: $F_{1, 13} = 6.35$, $p < 0.05$).

In terms of saccade amplitude, error saccades were shorter than correct saccades (Experiment 1: $F_{1, 23} = 166.68$, $p < 0.001$; Experiment 2: $F_{1, 13} = 125.34$, $p < 0.0001$) and there was a significant interaction between the accuracy of saccadic response and the category of the target stimulus (Experiment 1: $F_{1, 23} = 44.88$, $p < 0.001$; Experiment 2: $F_{1, 13} = 40.67$, $p < 0.001$). Pairwise comparisons showed that correct saccades toward face targets were larger than correct

saccades toward vehicle targets (Experiment 1: $p < 0.001$; Experiment 2: $p < 0.001$). However, error saccades were larger when the target was a vehicle (i.e. face distractor) than when it was a face (i.e. vehicle distractor; Experiment 1: $p < 0.001$; Experiment 2: $p < 0.01$).

Supplementary Table S3: Mean saccadic reaction times and amplitudes of saccades in the original data and when excluding "express" saccades falling below the 120 ms latency cut-off.

	**Mean Saccadic Reaction times (in ms)**				**Mean Amplitude (in degrees of visual angle)**			
	Target: Face		Target: Vehicle		Target: Face		Target: Vehicle	
	Correct	Error	Correct	Error	Correct	Error	Correct	Error
**Experiment 1**								
Original data	187	175	212	189	6.66	4.64	6	5.2
Excluding "express" saccades	190	181	216	191	6.76	4.79	6.11	5.35
**Experiment 2**								
Original data	207	194	233	206	6.75	4.61	6.26	5.7
Excluding "express" saccades	210	199	238	209	6.95	5.04	6.47	5.92

Overall, these analyses suggest that although likely present, express saccades represented a very small proportion of the data and were evenly distributed across conditions. Furthermore, they only had a limited impact on the reported results, which mainly reflected saccades executed in response to the visual stimuli.

References:

Agresti, A., & Caffo, B. (2000). Simple and Effective Confidence Intervals for Proportions and Differences of Proportions Result from Adding Two Successes and Two Failures. *The American Statistician, 54(4),* 280-288. https://doi.org/ 10.1080/00031305.2000.10474560.

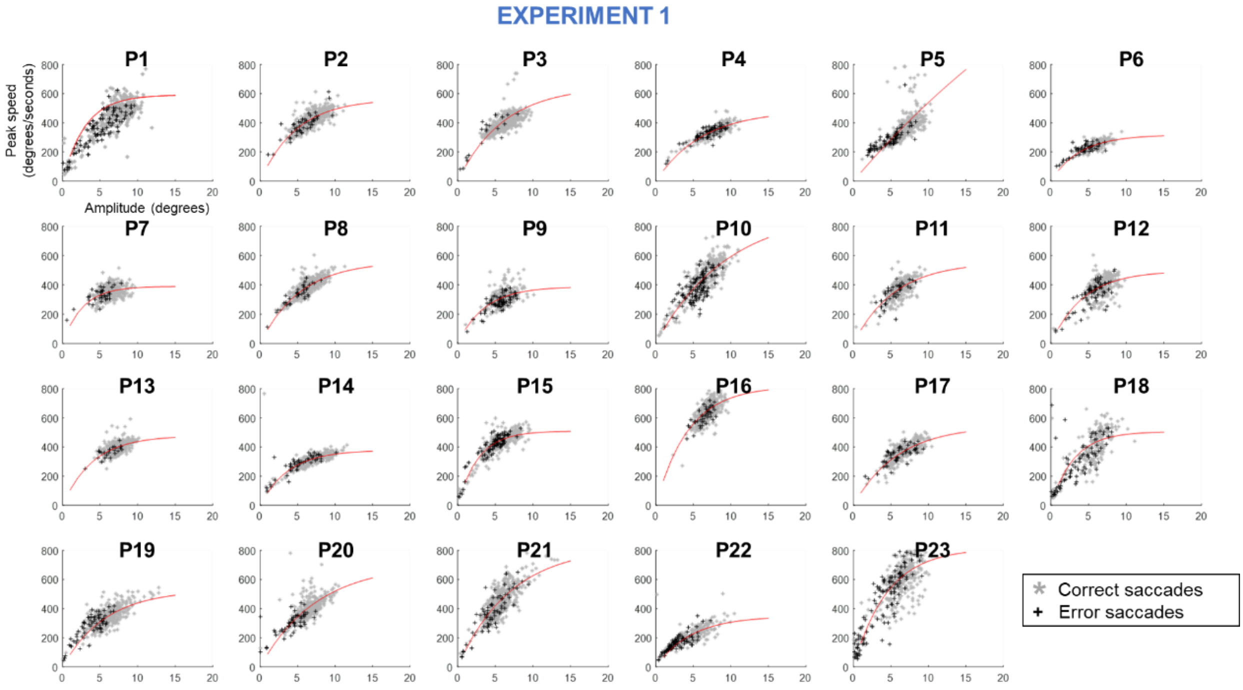

Supplementary Figure S2: Main sequence (peak speed as a function of amplitude) of correct and error saccade for each participant of Experiment 1.

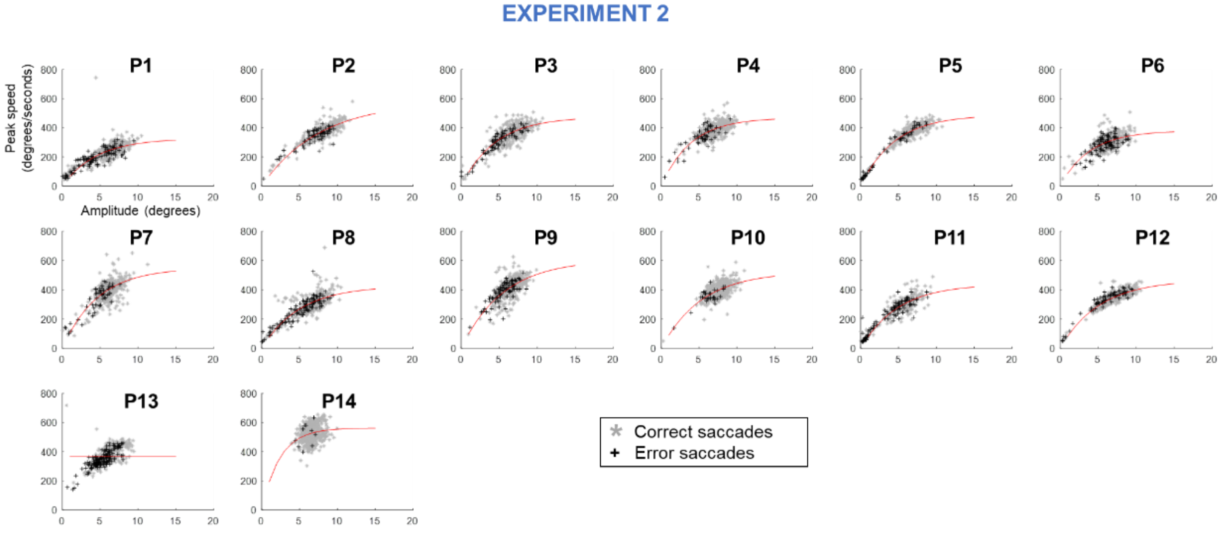

Supplementary Figure S3: Main sequence (peak speed as a function of amplitude) of correct and error saccade for each participant of Experiment 2.

Supplementary Data:

In order to test whether our results could be explained by a systematic bias in the programming of saccade amplitude toward faces, relative to vehicles, we performed a control experiment (N=13, 9F, mean age ± SD: 29 ± 7 years) with the same procedure as in Experiment 2, except that only one image (face or vehicle) appeared in the left or right visual field. Participants were simply instructed to make a saccade as fast as possible toward the white cross added in the center of the image. All images (120 faces, 120 vehicles) were the same as in Experiments 1 and 2 and were presented once in each visual field, resulting in 480 trials (the experimental session lasted about 40 minutes). Paired t-tests on the amplitude of correct saccades toward face and vehicles images revealed that saccades toward faces (mean ± SD: 6.76 ± 0.41°) were slightly but significantly larger than saccades toward vehicles (6.52 ± 0.51°, $t_{12} = 4.47$, $p < 0.001$). These results therefore suggest a differential programming of saccade amplitude according to the content of visual stimuli, with a tendency to make larger saccades toward face than vehicle stimuli, even in the absence of a distractor image. However, it can be noted that the difference in amplitude of saccade toward face and vehicle stimuli observed in this control experiment was much smaller (mean difference = 0.24°) than the differences observed in Experiment 1 (mean difference = 0.66°) and 2 (mean difference = 0.49°). In order to assess the significance of this reduced difference in our control experiment, relative to Experiment 2 (which was more comparable to the control experiment in terms of procedure and task), we performed an ANOVA on the amplitude of correct saccades, with the Category of the target image (Face, Vehicle) as a within-subject factor, and the Experiment (Experiment 2, Control Experiment) as a between-subject factor. We found a significant interaction between these two factors ($F_{1,25} = 4.41$, $p < 0.05$), suggesting that the difference in saccade amplitude toward face relative to vehicle stimuli was higher in Experiment 2 than in the Control Experiment. Interestingly, this interaction was driven by overall smaller saccades toward vehicles in Experiment 2 relative to the Control Experiment, while the amplitude of saccades toward faces remained relatively constant in both experiments. Overall, these results indicate that, although there is a general tendency to make smaller saccades toward vehicle than face stimuli, this effect was amplified by the presence of a face distractor, in line with our interpretation of a greater weight of face stimuli in interfering with a saccade program toward a vehicle than the other way around.
